# Reducing causes of inequity: policies focused on social determinants of health during generational transitions in Colombia

**DOI:** 10.1080/16549716.2017.1349238

**Published:** 2017-08-02

**Authors:** Juan Carlos Rivillas, Fabian Dario Colonia

**Affiliations:** ^a^ National Health Observatories, Department of Epidemiology and Demography, Ministry of Health and Social Protection, Bogota, Colombia; ^b^ Initiative Research in Health Services and Systems, School of Health, University of Valle, Cali, Colombia; ^c^ Trans-Sectoral Advisor /Technical Secretary of the Public Health Inter-Sectoral Commission, Ministry of Health and Social Protection, Bogota, Colombia

**Keywords:** social determinants of health, health inequalities, health in all policies, inter-sectorial coordination, generational transitions, Colombia

## Abstract

Health inequalities often result from social inequities, and those, in turn, are generated by social determinants of health (SDoH). Hence, to reduce health inequalities, it is necessary to consider all health-related determinants. Disadvantages arise even before birth, and they tend to accumulate throughout an individual’s life. Thus, policy actions intended to overcome these health inequalities should take place before birth and continue throughout life. This review aimed to describe the first steps of that Colombia has taken to reduce health inequalities during generational transitions through an inter-sectorial coordination upon SDoH. The review was guided by the question ‘What are the implemented policy responses aimed to reduce health inequalities during generational transitions in Colombia, and what can be considered in order to improve inter-sectorial coordination?’ Given the novelty of this area of research, the existent literature is presented more as narrative, rather than systematic review. Seven policies focused on five SDoH have been taken as examples. These policies show how Colombia plans to reduce health inequalities by acting upon these five SDoH: (1) early childhood development, (2) opportunities for education and first employment, (3) improved housing conditions, (4) social protection for families, and (5) vulnerable populations (e.g. elderly population). Additionally, more specific cases are examined in more detail that take place during sensitive periods in a person’s life, such as pregnancy, birth, early childhood, entry to higher education, first time job search, family building, elderly years, and so on. The evidence represents Colombian’s first steps toward reducing health inequalities during generational transitions. Elimination of health inequalities becomes feasible when governments recognize the importance of bringing opportunities to the worst-off populations, as well as the pivotal role of properly and well-coordinated inter-sectorial actions.

## Background

Health inequalities result from social inequities, which in turn are generated by the action of social determinants of health (SDoH) [1]. Therefore, in order to reduce inequalities in health, it is necessary to consider all health-related determinants known as conditions in which people are born, grow, live, work, and age [2]. These determinants include but are not limited to: genetic variations; place of living; relationships with family, friends, and colleagues; the immediate surrounding environment; transportation; urbanization; economic development; housing conditions; household incomes; educational attainment; life-style, and the access or use of health services. Thus, to reduce these inequalities, it is imperative that all government sectors and public entities work together to create a health equity-oriented approach by utilizing SDoH to make it possible [3].

Furthermore, such an approach can provide guidance to countries on the understanding of the concepts of health inequalities. It will also help to identify and to implement a framework for monitoring health inequalities [4]. Health inequity is a normative concept that cannot be accurately measured or monitored. Hence, monitoring health inequalities has been used as an indirect measure to assess the existence of health inequities between two or more social groups through the social scale [5]. However, it is worth mentioning that this framework is only one out of several approaches that can be used to address SDoH as well as monitoring health inequalities.

Five specific SDoH So are briefly discussed, as follows: (1) childhood development, (2) opportunities for education, (3) opportunities for first employment, (4) housing conditions, and (5) social protection for families and for vulnerable groups, including elderly populations.

1. Childhood development: The social and financial investment in early childhood development plays a significant role in reducing inequalities throughout an individual’s life. For instance, since 2000, there has been increasing recognition of early childhood development regarding physical, social, emotional, and linguistic-cognitive development. Proper childhood development represents a crucial determinant for lifetime opportunities, as well as having a good impact in an individual’s health [6]. Therefore, addressing early childhood development requires providing the best possible start for children, which in turn will allow them to improve their skills and capabilities, as well as to achieve better control over their own lives. Additionally, investing in childhood development will likely ensure the reduction of health inequalities in subsequent stages of their lives [7].
2. Employment clearly has a considerable impact on health as a social determinant. Hence, many researchers have consistently agreed that unemployment is harmful to health, in particular unemployment during youth [8,9]. Importantly, unemployment can also affect subsequent job opportunities and expectations [10]. Unemployment can cause an onset and or increment of alcohol consumption and smoking habits. Furthermore, families and communities can also experience poor health outcomes as a result of financial problems [8,11].
3. Poverty and social exclusion are two major determinants of health at the community, family, and individual level [2]. Children born into poor families are more likely to develop physical and mental-health problems as well as social struggles during their lifetime [3,4,12]. Families living in socially disadvantaged settings are more likely to suffer communicable diseases and injuries by external causes, such as traffic accidents, gender-based violence, or interpersonal violence [13].
4. Housing conditions may also influence health outcomes. For instance, inadequate housing conditions have a major impact on each of the family member’s physical and mental health [14,15]. They may also influence children’s educational achievements and increase the likelihood of engaging in risky health behaviours such as smoking, alcoholism, psychoactive substance consumption, and suicide attempts [16]. Lastly, when an individual lives in an overcrowded house, the risks of acquiring respiratory diseases, tuberculosis, meningitis, and stomach conditions increase dramatically [17,18].
5. Educational opportunities have consequences on health systems and heath outcomes at all levels, and in many cases, health is affected by a lack of these opportunities. In other words, a low educational level is associated with health risk behaviours, stress, depression, low self-esteem, and poor health outcomes [19], whereas a higher educational level may increase health outcomes because decisions about self-care and healthy life-styles tend to happen more often for those individuals [6].

Thus, SDoH are known to affect the overall population health in several ways [20]. This means that health problems should be addressed through policies that coordinate efforts across sectors, make efficient use of public resources, and consider the health impact of decisions made by non-health sectors [21]. Acknowledging such a need has resulted into a new approach that conveys inter-sectorial collaboration and policy making known as Health in All Policies (HiAP).

The HiAP approach aims to view health as a common outcome of all policies, which, in turn, requires multi-sectorial action. That is, if a policy addresses transportation, housing, education, nutrition, water, and sanitation, this will results in the good health of individuals. This framework of multi-sector action refers to the action between two or more sectors (e.g. health, transport, and the environment) within the government. It is also used as a synonym for inter-sectorial action [22,23].

As previously mentioned, disadvantages become evident even before birth, and they can accumulate throughout life. Therefore, it makes sense to see how actions to reduce health inequalities must also start before birth and continue through life. Such an early intervention can potentially help to break or can at least influence the linkage between early disadvantages and mortality results during life [2,6].

In 2010, the ‘Fair Society, Healthy Lives’ report recommended six policy goals aligned with the Commission of Social Determinants of Health (CSDH-WHO). These policies aimed to improve health equity with a life-course approach. The report focused on the specific social determinants mentioned above: early childhood development, employment opportunities; and social protection. The six policy goals are:

1. To give every child the best start in life;
2. To enable all children, youth, and adults to develop their capabilities and to have control over their own lives;
3. To create fair employment opportunities for all;
4. To ensure healthy standard of living for all;
5. To create and develop healthy and sustainable places and communities; and
6. To reinforce the role and impact of prevention.

Since 2012, Colombia has had an important leadership role in understanding SDoH. Colombia has also played an important role in the implementation of tools for monitoring inequalities in health and the promotion of the HiAP approach as recommended the CSDH-WHO [2,20].

The commitment to collaborative and participatory approaches in policy making has been possible because the current health policy makers recognize the importance of reaching a more inclusive society by setting cross-sectorial goals. Furthermore, the following publications show that various considerations have been implemented: (1) recommendations made in the call for action ‘Closing the Gap in a Generation’ [6]; (2) the recommendations made in ‘Fair Society, Healthy lives’ [7]; and (3) those added in the call for action ‘The Health Gap: The Challenges of an Unequal World’ [13].

In sum, these three publications describe ways to reduce and prevent health inequalities during an individual’s lifetime. They relentlessly highlight the importance of providing the best beginning for children from birth and enabling adolescents and adults to have access to good-quality education and job opportunities as the best way to empower them over their own lives. In short, at this stage, Colombia is moving from the theory to a more hands-on approach that aims to have long-term inclusive policies while at the same time advocating for multi-sectorial coordination.

Some important advances in this regard include the implementation of: (1) the *Decennial Public Health Plan 2012* *–* *2021*, with a special focus on SDoH [24]; (2) the National Health Observatory and the Observatory for Monitoring Health Inequalities (ODES Colombia) [25]; (3) the creation of an inter-sectorial CSDH-WHO (CISP) [26]; and (4) the design of tools to ensure the impact of national policies at the local and institutional levels, as well as the Health Equity Strategy (PASE a la Equidad en SALUD) [27].

In 2013, the Colombian government launched the *D* *ecennial Public Health P* *lan 2012* *–* *2021* as a political measure to reduce health inequalities, with three target recommendations: (1) to move toward ensuring effective access to the right to health; (2) to improve the living conditions that have a modifying impact on health and reduce the existing burden of disease; and (3) to keep zero tolerance toward avoidable mortality, morbidity, and disability.

Then, in 2014, the government launched ODES Colombia as an additional tool to measure, analyze, and explain health gaps, as well as to move forward regarding equity-oriented decision making. ODES Colombia provides useful information that may explain potential health disparities among people from different social groups. These disparities are also observed on the basis of poverty, vulnerability, discrimination, and a lack of access to health services [25]. The mission of the Observatory is to foster collaboration in order to develop and to adapt health equity policies aimed to bring those most marginalized and with less opportunities to acceptable levels through social scale.

The first consideration in implementing the HiAP approach is that Ministries of Health understand where health policy priorities are with respect to policies in other sectors. Nevertheless, it requires a willingness to work with cross-sectorial processes driven by other sectors and to provide information and guidance from the perspective of health. In 2015, the inter-sectorial CSDH-WHO (CISP) was established in order to ensure HiAP at the country level. This Commission recognized that while other sectors can serve the goals of health, health could significantly and collaboratively contribute to the goals of other sectors.

**Figure 1.**
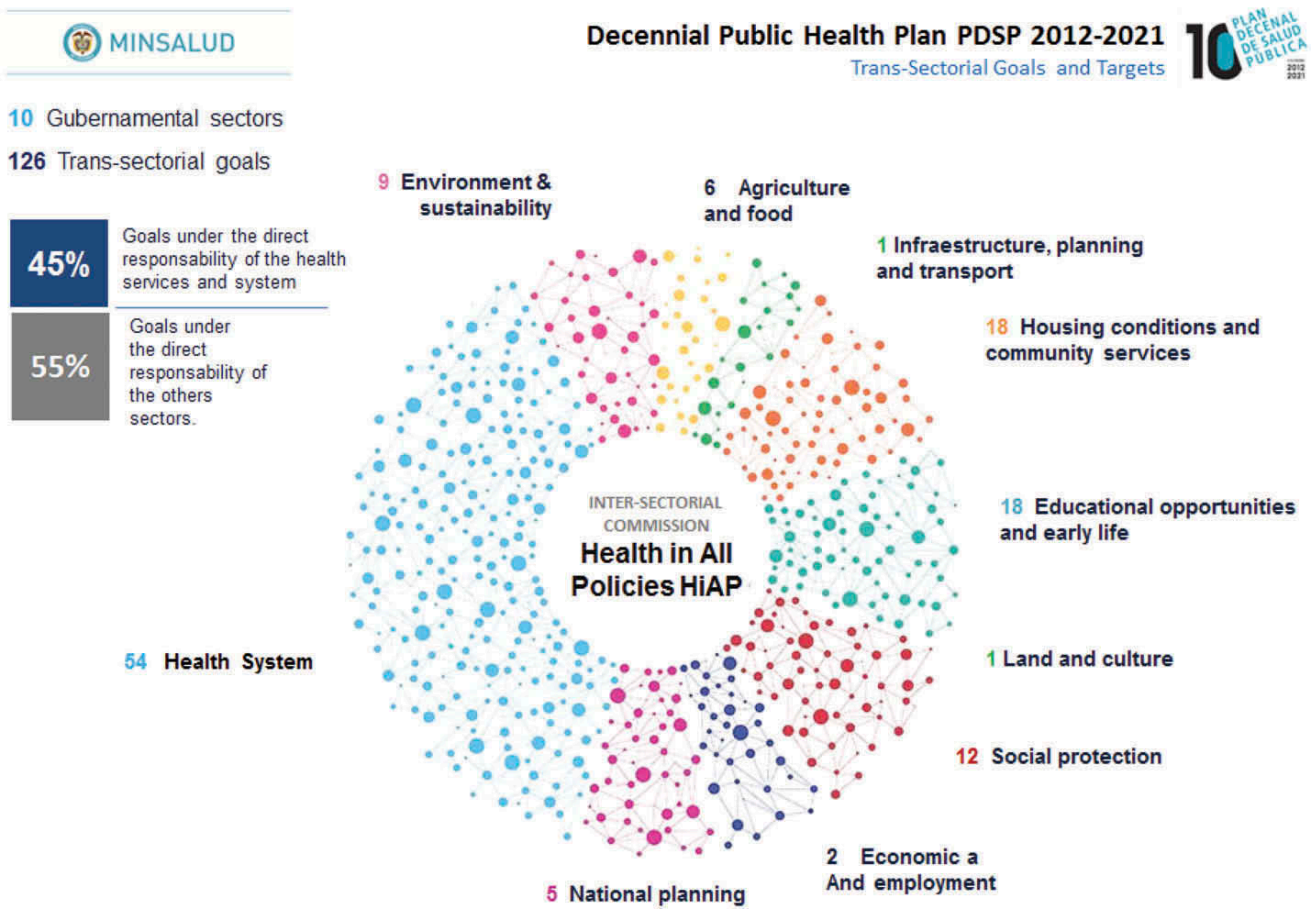
Colombian inter-sectorial Commission of Social Determinants of Health (CISP). Source: Built by the authors from the Colombian Inter-Sectorial Commission of Social Determinants of Health (CISP), Ministry of Health and Social Protection, Colombia, 2017.

Figure 1 shows the main elements of CISP. CISP has put together 118 inter-sectorial goals (represented by circles) from 11 several government sectors, included the *Decennial Public Health Plan 2012–2021*. For example, the health sector is near 55% of all goals and has an inter-sectorial nature, which means that other sectors such as education, employment, agriculture, the economy, social assistance, the environment, housing, and culture are needed to ensure the progressive realization of targets and goals by 2021, whereas 45% of goals are the direct responsibility of health services and systems.

Therefore, the objective of this article is to describe the first steps of that Colombia has taken to reduce health inequalities through an inter-sectorial coordination upon SDoH.

## Methods

The review was guided by the question ‘What are the implemented policy responses aimed at reducing health inequalities during generational transitions in Colombia, and what can be considered in order to improve inter-sectorial coordination?’ Given the novelty of this area of research, the existing literature is presented more as narrative, rather than systematic review.

Initially, a review was undertaken of the literature on implemented policies on five selected SDoH: early childhood development, employment, poverty measured through household incomes, housing conditions, and opportunities in education. These programs and policies were grouped per generational transitions: from pregnancy to early childhood, adolescence and youth (mainly during first job searches), adulthood (when building a family), and aging. The main interes was on responses focused on the most disadvantaged and vulnerable populations. At least one large implemented policy to reduce inequalities was identified according to social determinants. However, it was not possible to evaluate many qualitative or quantitative studies on this subject within the country because there was a lack of published work, despite the implementation of various programs.

We utilized Digital Institutional Repository data (RID) from the Ministry of Health and Social Protection using the following terms: sexual and maternal health policies, family policies, aging policies, SDoH, causes of inequities, equity-approach interventions, inequalities in health, and inter-sectorial coordination. RID is currently the most direct access to data on health sector within the country. The initial review was supplemented with specific searches using Google Scholar, and Google, and grey literature in the following areas: reduction of poverty, housing conditions, employment and unemployment, education policies, social inequalities in health, and causes of inequities.

## Results

Seven policy responses were identified addressing at least one of the five selected SDoH, which have been implemented during the last seven years.

**Figure 2.**
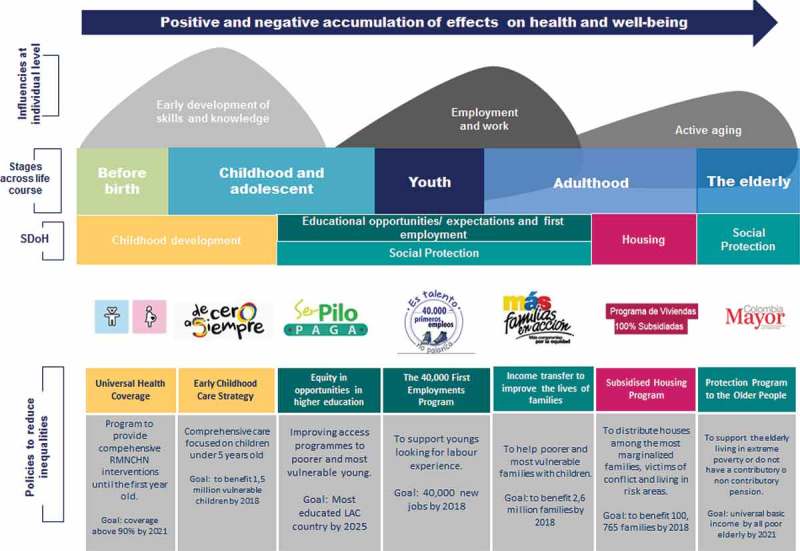
Seven policies to reduce social inequalities in health during generational transitions in Colombia, 2017. Source: Built by the authors.

Figure 2 shows the seven policies to reduce social inequalities in health during generational transitions in Colombia. This selection of policies focuses on different stages of the life course, and their potential accumulation of the effects on health and well-being at the individual level; particularly those among the poorest children and their families, and youth and the elderly with less opportunities. Among others, the policies include the progressive implementation of universal health coverage for children; improvement of the life and health of those teenagers and youth who are already attending school; young adults searching for their first job; and adults who have established a family on their own. It also includes the promotion of health services, the importance of well-being, and independence of the elderly.

## Discussion

### Early childhood development

First, the Safe Maternity Policy made it possible to deliver the best start for children, even before they were born, through the establishment of the right of access to healthcare improving the Sexual, Reproductive, Maternal, Newborns and Child Health (RMNCH) coverage [28]. This initiative is important because it exists at the constitutional level with a commitment at the presidency. As a result, in 2010, the country reported higher family planning indicators (>85%, even in rural and the most marginalized areas).

Second, the From Zero to Always Policy is an early childhood development initiative focused on the protection, nutrition, health services, and early education of poorer children [29]. The purpose of this policy targets children’s care from pregnancy to approximately five years of age. Currently, nearly 24% of children under the age of five have benefited from this comprehensive care policy. However, the challenge remains, as From Zero to Always has a great national goal to reach >1,500,000 children living in potentially risky environments. Nevertheless, an inter-institutional coordination aims to achieve 100% coverage by 2018.

### Opportunities for education and first employment

Colombia is planning to become the most educated Latin American country by 2025. Hence, strategies such as widening opportunities and access for children to attend schools and the provision of good and high-quality education become imperative, particularly among youth in the most disadvantaged settings. Importantly, this goal is critical for the realization and further development of the HiAP approach. Encouraging data show that in 2012, around 17,000 out of the best 27,000 scores reported in the high-school test ‘ICFES’ were achieved by students living in the most marginalized neighborhoods.

The political response was the implementation of the program called Ser Pilo Paga or Being Smart Pays Off [30]. This program was created to enhance quality, equity, and efficiency in higher education to students from the poorest families. This program improved the opportunities of youth by using an equity-oriented approach, and it also increased the availability of funds and scholarships to ensure access to academic programs in high-quality universities within the country in order to meet their academic expectations.

Fourth, to ensure youth access to the work marketplace, the government implemented the program 40,000 First Employments [31]. The first consideration for implementing this program was to recognize the difficulties that most marginalized youth often face when searching for jobs because many jobs require previous experience. Therefore, it was fundamentally an inter-sectorial coordination between labor and private and business sectors in order to ensure 40,000 new jobs by 2018. To achieve this goal, the government allocated a wide budget to the enterprises involved and incentivized them to hire young people for six months, after which the companies commit to hiring at least 60% of youth in the program. Importantly, those who did not get hired still gained the work experience and developed skills required for future job opportunities.

### Opportunities of affordable housing for poorer and vulnerable families

Fifth, the Subsidized Housing Program [32] was born in response to the situation of extreme poverty of thousands of households that were not able to access the granted credits for houses by the traditional mechanisms offered by the market. This program is intended to distribute 100,765 houses and to move forward toward a sustainable development goal that seeks to reduce poverty in Colombia by 2030. In this case, the challenge for the housing sector is the prioritization of the most vulnerable and marginalized families, particularly those who have been victims of armed conflict and forced displacement, those affected by natural disasters, and those living in risky areas.

### Social protection for the most vulnerable families and the elderly

A crucial aspect for inter-sectorial action in reducing inequalities is to recognize the importance of family within society. Mas Familias en Accion [33] is a program of conditioned transfers, which seeks to reduce extreme poverty and income inequity between households. The purpose of the initiative is the improvement of the living conditions of the poorest and most vulnerable families by complementing their household incomes with government aid. In return, families must encourage the education of their children and adolescents, and they must commit to attend the development controls of the children under the age of five. This program currently provides assistance to 2.6 million poor families in Colombia. Single mothers in rural areas also benefit from the Program.

Finally, Colombia’s Elderly Population program (Colombia Adulto Mayor) [34] was created to protect the elderly population from living in conditions of extreme poverty, mainly those who are homeless, experiencing loneliness, or those who do not have a contributory or non-contributory pension. This program currently benefits >1,258,000 elderly. Furthermore, every two months, the social protection sector provides a subsidy to cover basic needs and to contribute to the improvement of their quality of life. The goal of this policy is to achieve universal coverage for all elderly by 2021.

## Conclusions

Colombia is striving to establish inclusive policies that aim for a more inclusive society. It is doing so by targeting important life transitions among the most marginalized population who have fewer opportunities (i.e. children, youth, lower-income families, and the elderly). Moreover, these policies are meant to go hand-in-hand with universal health coverage, that is, providing full health coverage for family planning and during the first year of life. Equally important is access to the school system, subsequent access to higher education, and opportunities to acquire work experience. These policies are of intrinsic relevance when it comes to reducing inequalities among Colombian society.

Importantly, to prevent and reduce inequalities, there needs to be a joint perspective between education and the job industry. By interconnecting these important aspects of an individual’s life, as a society, healthier life transitions would be fostered for all.

The implementation of these equity-oriented responses also shows the commitment to putting poor and vulnerable families at the heart of the inter-sectorial agenda. However, among the challenges already identified for implementing HiAP, failure to place the concept of health as part of policy’s agenda was found. This is because in the past, health has not been considered an indispensable aspect of policy but rather as a separate sector alien from other sectors. Therefore, it is absolutely imperative to ensure that all sectors (i.e. education, job industry, the environment, climate change, etc.) understand the impact they have on health, as well as the positive implications of having health in all policies.

Moreover, further challenges and limitations can arise along HiAP, as described Diez et al. [34], for instance limitations achieving a productive multi-sectorial collaboration. This may include gaps between the written policy and the actual implementation of it. Other possible limitations are those inherent to each sector, for instance busy schedules, interests upon any given political current, and high levels of corruption to name a few. Other limitations could be the difficulty transferring experiences from a particular sector to the context of HiAP.

Lastly, this policy analysis is intended to facilitate the initial assessment of inter-sectorial coordination while addressing social inequalities in health during generational transitions in Colombia. As Colombia progresses along a learning path, the current work can be considered as evidence that demonstrates the importance of health within multiple sectors. Importantly, successful results from this type of policy are planned to be achieved by 2021, with some potential impacts by 2030 as well. Both deadlines require a great deal of commitment and rigorous evaluation. After all, as Peter Goldblatt recently mentioned in one of his speeches at the Health Equity Assessment Toolkit HEAT (WHO/PAHO on December 2015 in Chile) ‘where there are policies, at least something can be done’.

## Paper context

To reduce health inequalities, it is necessary to consider all health-related determinants. Policy actions intended to overcome these health inequalities should take place before birth and continue throughout life. This review describes the importance of maintaining the reinforcing inter-sectorial coordination. Seven existing inter-sectorial policies focused on five SDoH have been taken as examples. The evidence represents Colombian’s first steps toward the reduction of health inequalities during generational transitions through proper inter-sectorial coordination.
